# Long-Distance Interdisciplinarity Leads to Higher Scientific Impact

**DOI:** 10.1371/journal.pone.0122565

**Published:** 2015-03-30

**Authors:** Vincent Larivière, Stefanie Haustein, Katy Börner

**Affiliations:** 1 École de bibliothéconomie et des sciences de l’information, Université de Montréal, Montréal, Canada; 2 Observatoire des sciences et des technologies, Centre interuniversitaire de recherche sur la science et la technologie, Université du Québec à Montréal, Montréal, Canada; 3 Cyberinfrastructure for Network Science Center, School of Informatics and Computing, Indiana University, Bloomington, Indiana, United States of America; Katholieke Universiteit Leuven, BELGIUM

## Abstract

Scholarly collaborations across disparate scientific disciplines are challenging. Collaborators are likely to have their offices in another building, attend different conferences, and publish in other venues; they might speak a different scientific language and value an alien scientific culture. This paper presents a detailed analysis of success and failure of interdisciplinary papers—as manifested in the citations they receive. For 9.2 million interdisciplinary research papers published between 2000 and 2012 we show that the majority (69.9%) of co-cited interdisciplinary pairs are “win-win” relationships, i.e., papers that cite them have higher citation impact and there are as few as 3.3% “lose-lose” relationships. Papers citing references from subdisciplines positioned far apart (in the conceptual space of the UCSD map of science) attract the highest relative citation counts. The findings support the assumption that interdisciplinary research is more successful and leads to results greater than the sum of its disciplinary parts.

## Introduction

Long-distance relationships are tough. Scholarly collaborations across disparate scientific disciplines are even more challenging: Collaborators are likely to have their offices in another building, attend different conferences, and publish in other venues; they might speak a different scientific language and value an alien scientific culture. Over the last 20 years, interdisciplinarity has remained a hot topic in science policy [1–3]. Interdisciplinary is formed through problems, it is thus aimed at application, and can be defined as the combination of methods, theories and data of distinct disciplines to ideally derive a result that is greater than the sum of its parts [4]. As it is often taken for granted that it is a fruitful enterprise, governments and international organizations have pushed towards interdisciplinary research [5,6]. However, previous research provided conflicting evidence on its effect on the impact of research. For instance, at the level of research groups, Rinia, van Leeuwen and van Raan showed, using the percentage of papers published outside of physics as a measure of Dutch physicists’ interdisciplinarity, that interdisciplinary programs had lower impact (raw citations and raw impact factors) than programs that were at the ‘core’ of physics [7]. Applying relative impact indicators, the differences in impact between the groups were, however, lower. At the level of journals, Levitt and Thelwall provided evidence that, in the natural and medical sciences, papers published in journals to which more than one disciplinary classification had been assigned obtain fewer citations than papers published in strictly disciplinary journals [8]. Adams, Jackson, and Marshall calculated for 37,000 papers from two UK universities, the percentage of references made to journals published in disciplines other than that of the citing paper and found that interdisciplinary papers were not cited more than the ‘average’ paper, and that highly-cited papers were not the most interdisciplinary ones [9]. Also at the level of individual papers and using the percentage of references made to journals of a different discipline, Larivière and Gingras showed that, for some disciplines—such as biomedical research—an increase in interdisciplinarity was associated with a decrease in citations [10]. For some other disciplines, a moderate interdisciplinarity was associated with an increase in impact. In all disciplines, however, papers with very high or very low interdisciplinarity obtained fewer citations; a finding which has recently been corroborated by Yegros-Yegros et al. at the level of research groups [11]. Larivière and Gingras’ [10] results also show that the relationship between interdisciplinarity and impact was “highly determined by the citation characteristics of the disciplines involved, as articles citing citation-intensive disciplines are more likely to be cited by those disciplines and, hence, obtain higher citation rates than are articles citing non-citation-intensive disciplines” (p. 131). This has important consequences on the interpretation given to the relation between citations and interdisciplinarity, as it shows that the disciplines involved and their citation dynamics can influence the scientific impact of interdisciplinary relationships [10]. Recent work by Uzzi et al. rates the novelty of journal pairs in reference lists equate it with a possible novelty of results presented in the paper, and correlate it with the ultimate citation-impact of papers [12]. They find that novel combinations, when interjected with otherwise conventional combinations, yield increases in impact. Similarly, Klavans and Boyack [13] showed, using clusters of papers rather than disciplines, that the impact of citing papers increases with the topical distance of the papers they cite.

Despite these numerous analyses, the effect of long-distance interdisciplinarity on the impact of research remains open to debate. Similarly, while the relationships between disciplines have been extensively mapped and visualized [14–16], these are typically made to assess their intensity rather than their effect on the scientific impact (citations) of citing papers. This paper, thus, aims to measure and visualize subdiscipline distances and linkages to answer the following questions: What are the (sub)disciplines that increase impact when cited together? To the opposite, what are the interdisciplinary combinations that are associated with a decrease in citations? The visualization of these interdisciplinary combinations and their effect on the impact of papers will shed light on the network of (sub)disciplines that have a positive effect on the impact the papers citing them. This analysis is limited to the scientific impact of interdisciplinary research, as measured by citations received. Other types of impacts which interdisciplinary research may have on education, industry practice or society, health or the economy at large are not measured here. The results nonetheless shed light on the impact of interdisciplinary relationships in the scientific community, and may trigger more analyses—either quantitative or qualitative—on what it is exactly in these combinations of disciplines that result in highly cited research. This, in turn, can have significant implications for research policy and institutional planning.

## Results

### Number of subdisciplines cited and citation rates

**Fig 1:**
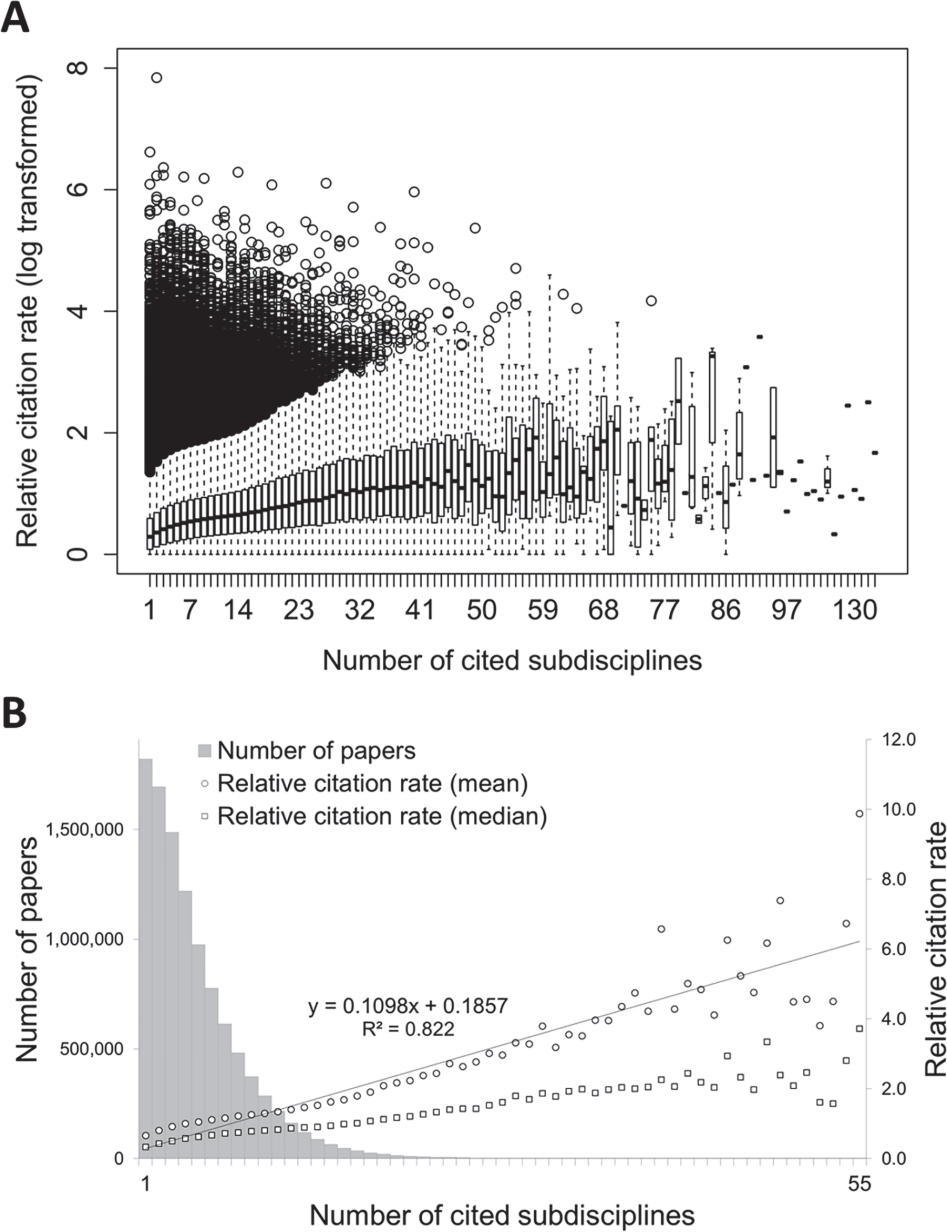
Number of papers and relative citation rates of papers that cite between 1 and 55 subdisciplines.

The relative citation rate—both in terms of average and media—of papers increases with the number of subdisciplines they cite, supporting the perceived success of interdisciplinarity (Fig 1, panels A and B). At citation impact below the expected number of citations of papers published in the same subdiscipline at the same year, strictly disciplinary papers, i.e., those citing papers from one subdiscipline only, obtain the lowest citation rates (0.66). These disciplinary papers make up 17.2% of all papers in the WoS. Papers citing 3 subdisciplines or less obtain a mean impact below world average (47.3% of all papers), while papers citing 4 subdisciplines (11.5% of all papers) obtain a mean citation rate that is on a par with the mean impact of papers published the same year in the same subdiscipline. On average, citation rates exceeding the world average of 1 are obtained by papers citing references from at least 5 different subdisciplines (41.2% of all papers). A similar trend is observed for the median—although the impact values are lower, as they are less likely to be influenced by a few highly cited papers.

### Citation impact of interdisciplinary combinations

**Fig 2:**
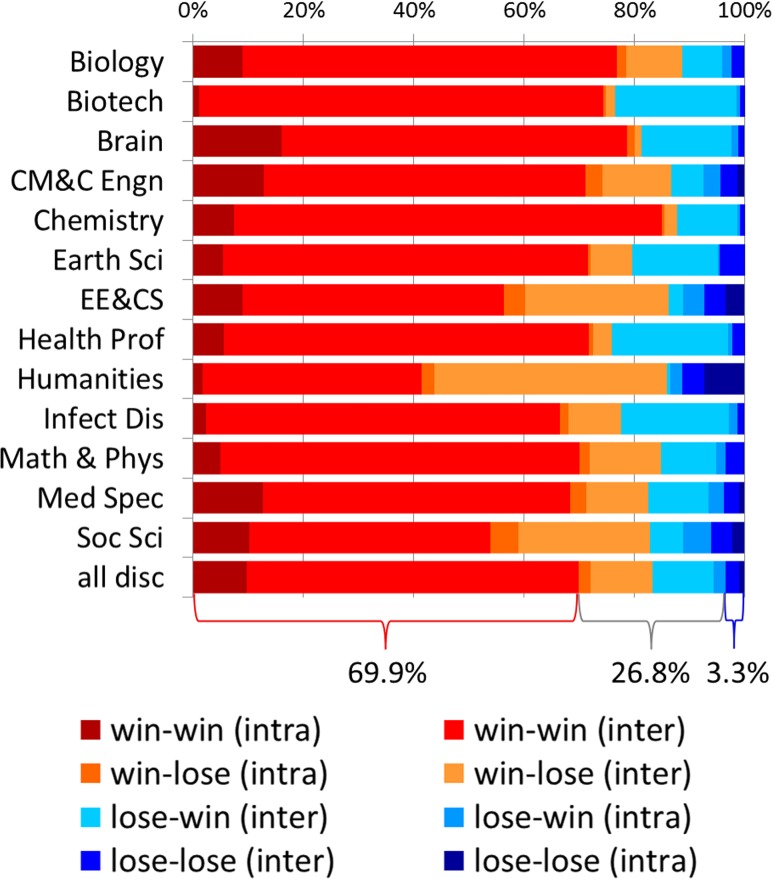
Percentages of inter- and intradisciplinary win-win, win-lose, lose-win and lose-lose relationships of subdiscipline pairs.

The mean relative citation rate of all interdisciplinary subdiscipline pairs included in this study is 1.54. Out of all 161,994 interdisciplinary subdiscipline pairs, 69.9% are win-win relationships, 26.8% increased impact for one of the subdisciplines, and only 3.3% do not exceed expected citation rates in any of the two subdisciplines (Fig 2). Note that the majority of interdisciplinary papers are win-win—this is due to the fact that strictly disciplinary pairs typically have an impact below average, as well as to the various interdisciplinary pairs having different numbers of papers. With 85% win-win pairs, interdisciplinary relationships by and with subdisciplines from *Chemistry* are most often beneficial, followed by *Brain Research* (78.8%) and *Biology* (76.9%). Most lose-lose relationships occur with subdisciplines in the *Humanities* (11.2%) and *Electrical Engineering & Computer Science* (7.3%). From the perspective of a particular discipline, the *Humanities* are by far benefiting most from interdisciplinary pairs but other disciplines involved frequently lose, i.e., 44.5% of all *Humanities* subdisciplinary pairs are win-lose combinations. In terms of beneficial combinations, interdisciplinary co-citations that include subdisciplines from *Biotechnology*, *Chemistry* and *Brain Research* are most often successful as respectively 97.0%, 96.4% and 96.4% of all subdisciplinary combinations are either a win-win or lose-win, i.e., the citation impact of both or of the co-cited subdiscipline increased. Distinguishing between intradisciplinary, i.e., pairs of two subdisciplines belonging to the same discipline, and truly interdisciplinary pairs, the most interdisciplinary win-win pairs are in *Chemistry* (77.7%) and *Biotechnology* (73.2%), while *Brain Research* (16.0%), *Chemical*, *Mechanical*, *& Civil Engineering* (12.9%) and *Medical Specialties* (12.6%) show the highest percentages of intradisciplinary win-win pairs.

### Citation impact and topical distance

The mean relative citation rate of co-cited interdisciplinary pairs constantly increases with the distance between them (Table 1 and Table 2). Category A, which contains co-cited subdiscipline pairs closest to each other in the UCSD map—in fact 71.4% of the pairs in this category were assigned to the same discipline—has both the lowest citation rate (1.26) and lowest share of win-win pairs (62.4%), while category J, which at a mean distance of 205 contains the topically least related subdiscipline pairs was on average cited 72% above average. Except for distance categories A and J, the percentages of win-win relationships are quite stable at around 71%. This suggests that the increase in citation impact is not caused by an increase in the number of win-win pairs but rather by an actual growth of relative citation impact as distance increases. In other words, the probability of an interdisciplinary relationship increasing a paper’s relative citations in relation to both subdisciplines is similar across distance groups B to J but impact intensifies with greater distance. However, both medians (1.48–1.50) and standard deviations (0.79 to 0.97) of categories G to J suggest that the increasing citation rates in these four groups are less due to an overall increase but mainly due to a few highly cited pairs (Table 2).

**Table 1:** Number of interdisciplinary pairs per discipline, mean distance and mean, standard deviation and ratio of distant (F-J) and close (A-E) subdiscipline pairs of relative citation rate.

	** **	**Number of subdiscipline pairs**		** **	**Relative citation rate**		
**Discipline**		N	mean distance		mean	standard deviation	ratio of distant & close pairs
Biology		14,208	128		1.49	0.50	1.11
Biotech		4,738	110		1.29	0.42	1.03
Brain		24,466	99		1.35	0.54	0.99
CM&C Engn		18,371	106		1.75	0.84	1.24
Chemistry		11,888	121		1.47	0.52	1.01
Earth Sci		6,677	139		1.40	0.71	1.08
EE&CS		12,349	100		1.92	1.16	1.31
Health Prof		10,470	102		1.32	0.52	1.02
Humanities		1,957	127		2.46	1.72	2.03
Infect Dis		9,002	106		1.34	0.49	1.00
Math & Phys		8,238	120		1.76	1.01	1.22
Med Spec		21,301	105		1.46	0.66	0.97
Soc Sci		18,329	107		1.64	0.90	1.42
**All disciplines**		**161,994**	**110**	** **	**1.54**	**0.78**	**1.13**

**Table 2:** Distance categories of subdiscipline pairs (A-J) with mean distance and number of pairs, mean, standard deviation, median, minimum and maximum of relative citation rate and the percentage of win-win, win-lose/lose-win and lose-lose relationships per group.

**Distance group**	**Mean distance**		**Numberof pairs**	** **	**Relative citation rate**					** **	**Percentage of win vs. lose relationships**		
					mean	st. dev.	median	Min	max		win-win	win-lose &lose-win	lose-lose
A	17		15,790		1.26	0.45	1.20	0.11	13.81		62.4%	31.5%	6.1%
B	38		16,584		1.38	0.52	1.30	0.09	13.87		70.3%	25.9%	3.8%
C	58		16,404		1.45	0.64	1.34	0.24	13.64		70.5%	25.9%	3.6%
D	77		15,854		1.49	0.79	1.36	0.27	24.78		69.0%	28.0%	3.0%
E	96		15,882		1.51	0.80	1.38	0.13	27.53		69.7%	27.7%	2.6%
F	119		16,284		1.58	0.75	1.44	0.21	15.07		71.4%	26.0%	2.6%
G	141		16,160		1.64	0.79	1.48	0.25	12.08		71.2%	26.4%	2.4%
H	162		16,308		1.66	0.82	1.49	0.22	11.47		70.2%	27.1%	2.6%
I	182		16,358		1.71	0.96	1.50	0.23	27.22		71.0%	26.1%	2.9%
J	205		16,370		1.72	0.97	1.50	0.26	19.71		73.0%	23.0%	3.9%
**All pairs**	**110**	** **	**161,994**	** **	**1.54**	**0.78**	**1.38**	**0.09**	**27.53**	** **	**69.9%**	**26.8%**	**3.3%**

**Fig 3:**
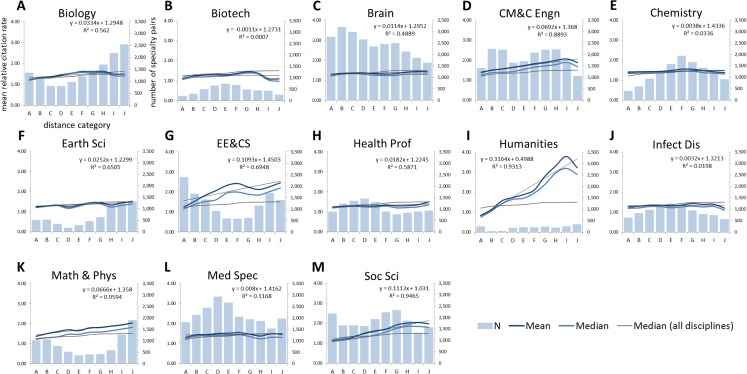
Number, mean and median relative citation rate of subdiscipline pairs across distance categories per discipline.

Fig 3 shows the number of co-citing papers as well as the relative citation rates across distance categories A to J for each of the 13 disciplines. Relative citation rates across distance categories increase linearly for the *Humanities*, the *Social Sciences*, *Chemical*, *Mechanical*, *& Civil Engineering* as well as *Math & Physics*, while some fluctuations can be observed in *Electrical Engineering & Computer Science*, *Biology*, *Earth Sciences* and *Biotechnology*. For example, in *Electrical Engineering & Computer Science*, relative citation rates increase from category A (1.25) to F (2.42), after which they show a decrease in G to I to reach 2.46 in J. *Earth Sciences* also shows a tendency of increasing citation impact with distance to the co-cited subdiscipline from 1.27 in distance category A to 1.52 in J with the exception of impact dropping in D (1.24) and H (1.34). *Brain Research*, *Infectious Diseases*, *Medical Specialties*, *Health Professions* and *Chemistry* show only a small increase of citation rates. It should, however, be noted that for none of the 13 disciplines impact decreases with increasing distance.

Table 3 shows the number and percentages of win-win and lose-lose relationships of interdisciplinarity pairs in the UCSD map of science. Thus it highlights where the most successful and unsuccessful combinations of co-cited subdisciplines are located in the landscape of scientific disciplines. Relationships which involve one winner and one loser (win-lose, lose-win) are not shown. On average, the percentage of win-win relationships is highest in *Chemistry* (mean: 85.0%; std. dev.: 6.9%) and *Brain Research* (78.4%; 12.6%) and lowest in the *Humanities* (35.0%; 23.0%) and *Social Sciences* (50.4%; 13.9%), which is likely a consequence of the lower citation density of those disciplines. On the lose-lose side, *Nursing Theory* (*Medical Specialties*) was involved in the most lose-lose relationships, both in absolute (62 lose-loses) and relative (19.6%) terms and the *Humanities* (23.7%; 26.5%) are involved in the most lose-lose relationships, on average. Subdisciplines associated with *Biotechnology* had, on average, the lowest share of lose-lose pairs (1.1%; 1.1%), which clearly suggests that its multiple application to life sciences, pharmacology, agriculture or engineering is beneficial to all subdisciplines involved.

**Table 3:** Number, mean number and mean percentage of win-win and lose-lose relationships per subdisciplines for 13 disciplines.

		win-win relationships							lose-lose relationships					
Discipline		N	# per subdiscipline			% of subdisciplines			N	# per subdiscipline			% of subdisciplines	
			mean	st. dev.		mean	st. dev.			mean	st. dev.		mean	st. dev.
Biology		10,921	254	80.8		75.6%	11.2%		326	8	6.3		2.6%	2.3%
Biotech		3,522	320	43.5		75.0%	12.6%		36	5	4.6		1.1%	1.1%
Brain		19,276	279	60.1		78.4%	11.1%		259	4	3.1		1.5%	2.2%
CM&C Engn		13,077	177	99.7		68.0%	18.4%		785	11	7.2		5.4%	4.2%
Chemistry		10,108	316	73.7		85.0%	6.9%		102	4	3.9		1.4%	2.1%
Earth Sci		4,788	228	64.1		71.2%	10.0%		295	14	9.6		4.9%	4.6%
EE&CS		6,973	127	77.7		53.8%	15.7%		905	16	10.2		8.1%	4.5%
Health Prof		7,512	268	63.3		71.5%	9.4%		228	8	8.0		2.5%	2.5%
Humanities		812	39	52.3		35.0%	23.0%		220	9	7.1		23.7%	26.5%
Infect Dis		5,989	260	74.6		65.4%	13.1%		106	6	4.8		1.9%	2.3%
Math & Phys		5,780	206	106.4		66.1%	16.5%		276	10	8.5		3.6%	2.4%
Med Spec		14,581	211	91.2		65.8%	16.7%		782	12	8.7		4.3%	3.2%
Soc Sci		9,889	143	82.0		50.4%	13.9%		1,104	16	9.2		6.9%	4.8%
All disciplines		113,228	209	104.4		65.9%	18.6%		5,424	11	8.7		5.3%	8.1%

### Mapping interdisciplinarity

**Fig 4:**
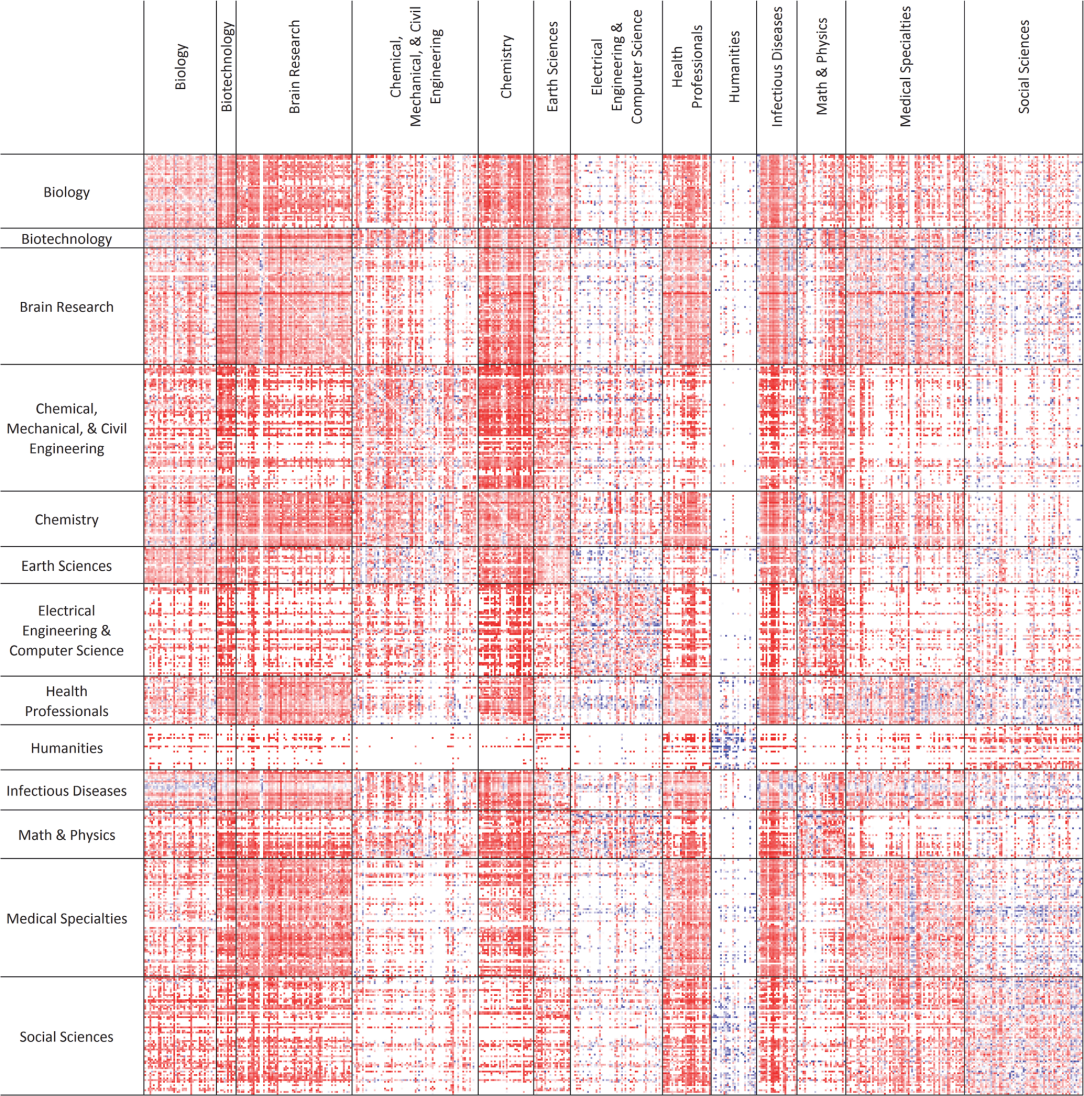
Heat map showing relative citation rates of subdiscipline pairs based on at least 30 co-citing papers, subdisciplines are grouped by superordinate discipline. Cells of subdiscipline pairs below the threshold of 30 co-citing papers and citation rates close to the world average are colored in white, those below in shades of blue (cold) and those above world average in shades of red (hot).

The heat map (Fig 4) visualizes the citation impact of all 80,997 occurring subdiscipline pairs *s*_*1*_ *-s*_*2*_ from the perspectives of both *s*_*1*_ (row) and *s*_*2*_ (column). For example, the majority of red cells in the *Social Sciences* (row) > *Brain Research* (column) field indicate that these interdisciplinary connections are mostly beneficial relative to expected citation rates in the *Social Sciences*, while the large number of blue cells in the *Brain Research* (row) > *Social Sciences* (column) field shows that from the perspective of *Brain Research*, many of these pairs do not result in high citation impact. The majority of red cells in the matrix reflects the overall success of interdisciplinary pairs. The heat map also highlights where interdisciplinary relationships do not deviate much from expected citation rates or do not appear at all (white color coding), for example between the *Humanities* and *Chemical*, *Mechanical*, *& Civil Engineering*.

**Fig 5:**
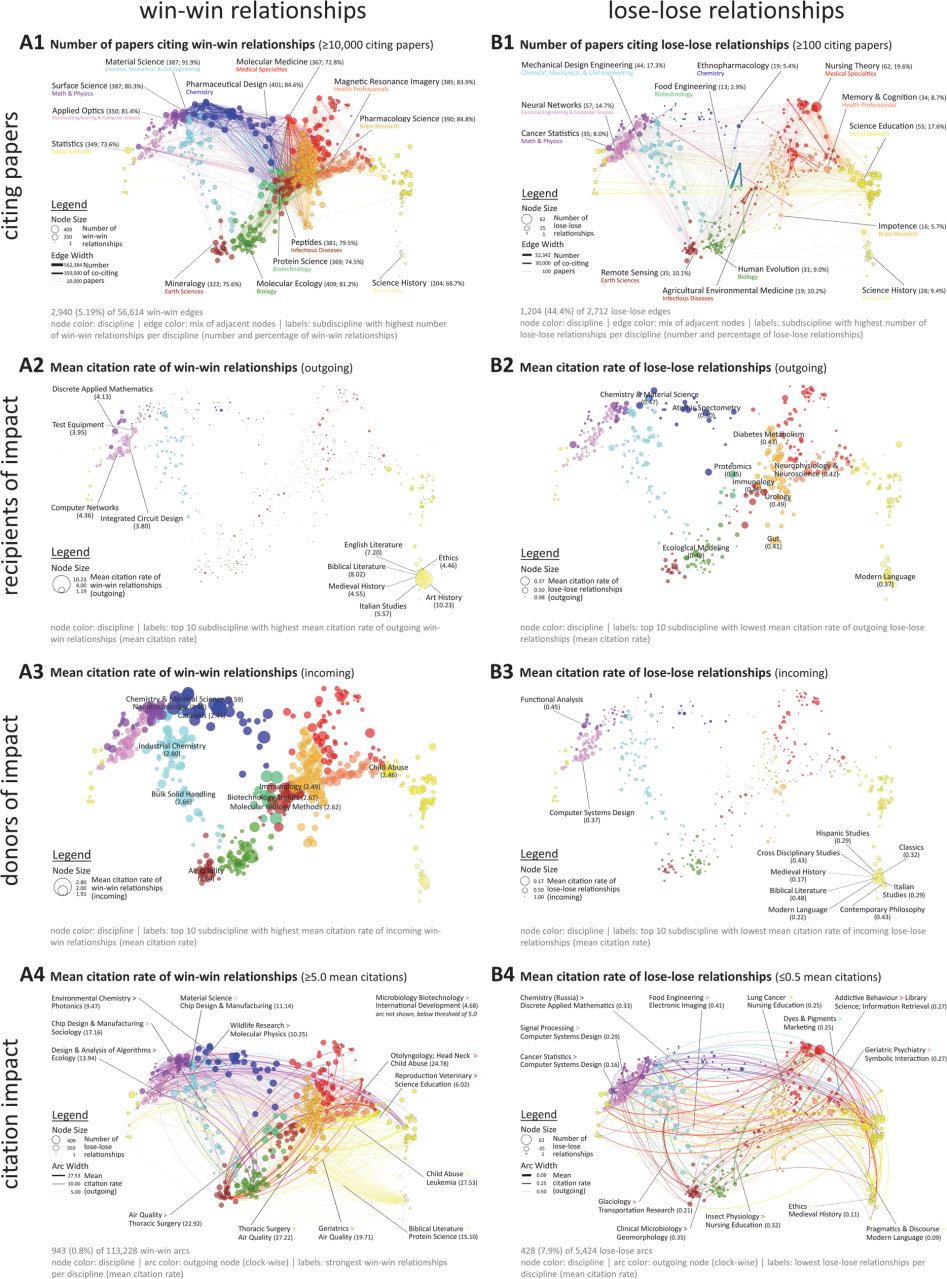
UCSD map overlay of win-win (A1-A4) and lose-lose (B1-B4) interdisciplinary combinations.

Fig 5 locates and highlights the strongest win-win and lose-lose linkages using the UCSD map of science and classification as a reference system [18] (see Supplementary Material). Relationships which involve one winner and one loser (win-lose, lose-win) are not shown. The number of papers citing win-win and lose-lose combinations are shown as edges in A1 and B1, respectively. As to be expected and emphasized by the strongest edges in A1, interdisciplinary subdiscipline pairs occurring most frequently are close to each other and mostly belong to the same or neighboring disciplines (color-coded). The most frequently co-cited win-win pair is *Protein Science* (*Biotechnology*) and *Clinical Cancer Research* (*Brain Research*), which occurred in 562,384 citing papers and focuses, among other topics, on the genetic basis of cancer. Lose-lose relationships appear much less frequently and often between subdisciplines belonging to distant disciplines. The most frequently occurring lose-lose pair is *Food Engineering* (*Biotechnology*) and *Food Chemistry* (*Chemistry*) (52,342 papers cite works from these two disciplines). The node size in A1 and B1 indicates the number of win-win and lose-lose relationships for particular subdisciplines. The “biggest winners” and “biggest losers” of interdisciplinarity are labeled for each discipline. Comparing A1 and B1, it can be seen that the number of wins are less skewed and more equally distributed across the science map than the number of losses. The subdiscipline with the most win-win relationships in absolute numbers is *Molecular Ecology* (*Biology*; 409 win-wins, 81.2% of all its interdisciplinarity relationships). However, *Material Science* (*Chemical*, *Mechanical*, *& Civil Engineering*; 387; 91.9%) obtained more win-wins as a percentage of all its interdisciplinarity combinations (Table 3).

Node size in A2/B2 and A3/B3 indicates the mean citation rate of all outgoing (A2/B2) and incoming (A3/B3) pairs. As such, the maps show which subdisciplines gain and lose. While the mean of outgoing citation links (mean of *s*_*1*_ *-s*_*n*_ for *s*_*1*_) highlights recipients of impact, the mean of incoming links (mean of *s*_*n*_ *-s*_*1*_ for *s*_*1*_) marks the donors, who provide most positive (win-win) or negative (lose-lose) impact to the co-cited subdiscipline. As shown in A2, the greatest recipients of win-win relationships belong to the *Humanities*, *Electrical Engineering & Computer Science* and *Math & Physics*, with *Art History* (mean incoming relative citation rate: 10.23) and *Biblical Literature* (8.02) benefiting the most. Donors are more equally distributed and appear in different disciplines with *Industrial Chemistry* (2.80), *Biotechnology Trends* (2.67) and *Bulk Solid Handling* (2.66) providing on average the highest citation rates to the co-cited subdiscipline. On the lose-lose side, the picture on the disciplinary level is reversed: Recipients of highest lose-lose relationships are equally distributed with *Modern Language* (mean incoming relative citation rate: 0.37 = 63% below expectations), *Gut* (0.41), *Neurophysiology & Neuroscience* (0.42) and *Diabetes Metabolism* (0.43) losing most. Donors of lose-lose, i.e., negative impact, are mostly concentrated in the *Humanities*, *Electrical Engineering & Computer Science* as well as *Math & Physics* with *Medieval History* (0.17) and *Modern Language* (0.22) losing most when co-cited with another subdiscipline.

Maps A4 and B4 highlight the strongest connections between two subdisciplines in terms of relative citation rates. In A4, arcs with a citation rate of 5.0 and higher are shown, while B4 depicts citation impact of 0.5 and lower, i.e., 50% below expectations. Node size indicates the number of win-win and lose-lose relationships and is thus identical to A1 and B1. Arcs move clock-wise from source to target and are colored according to the source. For each discipline, the strongest outgoing connection is labeled. The highest relative citation rate is obtained by *Child Abuse* when co-cited with *Leukemia* (27.53) and the lowest impact by *Pragmatics & Discourse* when co-cited with *Modern Language* (0.09). It can be seen that the strongest win-win combinations (links) are between the *Social Sciences* to *Brain Research* (e.g., *Child Abuse* to *Leukemia*, 27.53), *Brain Research* to *Earth Sciences* (e.g., *Thoracic Surgery* to *Air Quality*, 27.22), *Social Sciences* and *Medical Specialties* (e.g., *Child Abuse* to *Otolyngology*, *Head Neck*, 22.98), and *Medical Specialties* to *Earth Sciences* (e.g., *Otolyngology*, *Head Neck* to *Air Quality*, 17.08) and *Brain Research* (e.g., *Psychiatric Nursing* to *Vascular Surgery*, 15.36), while the strongest lose-lose links are within the *Social Sciences* and *Humanities* (e.g., *Pragmatics & Discourse* to *Modern Language*, 0.09 or *Ethics* to *Medieval History*, 0.11) but also range across the network such as from *Earth Sciences* (*Glaciology*) to *Chemical*, *Mechanical*, *& Civil Engineering* (*Transportation Research*, 0.21) or *Chemical*, *Mechanical*, *& Civil Engineering* (*Dyes & Pigments*) to *Social Sciences* (*Marketing*, 0.25).

## Discussion and Conclusion

Since the 1980s, interdisciplinary relationships have increased [17], likely because of “government programs and discourses promoting interdisciplinarity as a good thing in itself” (pp. 197–198). Our results provide evidence that, on the whole, interdisciplinarity research, as measured by co-citation links, is beneficial, as the majority of co-cited interdisciplinary pairs benefit in terms of citation impact, and as few as 3.3% of represent losses for both subdisciplines involved. While most win-win relations are associated with disciplines related to medicine, most lose-lose relationships are associated with disciplines that typically have lower citation rates, such as computer science and engineering, social sciences and the humanities. This might also be due to the lower coverage of literature published in these domains, which often appears in non-journal literature (conference proceedings, books, etc.).

Topical distance has a positive effect on scientific impact in the natural sciences and social sciences and to a lesser extent in medical disciplines. This might be caused by several factors, two of which are discussed here. First, combining methods and perspectives from multiple disciplines might be able to solve scientific problems or social issues that are more complex and that one discipline alone cannot solve [4]. Second, different subdisciplines have diverse intrinsic citation dynamics. As shown by Larivière and Gingras [10], papers’ citation rates are influenced by the disciplines they cite: a humanities paper citing papers in medicine is likely to obtain higher-than-average citations compared to other humanities papers, while a medical paper citing papers in humanities is likely to experience the opposite faith. This would explain why the humanities, as well as other low citation count disciplines, are more often involved in lose-lose and win-lose relationships.

## Materials and Methods

Disciplines and subdisciplines were assigned to journals using the UCSD map and classification system of science [18]. This classification covers 10 years (2001–2010) of WoS data and 8 years (2001–2008) of Scopus data and fractionally matches about 25,000 journal and other source titles to 13 disciplines and 554 subdisciplines using a combination of bibliographic coupling and keyword co-occurrences as similarity measures on journal level. The distance between two subdisciplines is calculated using the x-y positions of the 554 subdisciplines on the UCSD map. Note that this map wraps around a cylinder, i.e., the left most nodes are connected to the nodes on the far right. The cylinder circumference is 624 and the maximum distance is half of this: 312. The distances for the 153,181 subdiscipline pairs range from 0.63 to 294.32.

This study uses a definition of interdisciplinarity based on cited references. An interdisciplinary relationship between 2 of the 554 UCSD subdisciplines exists, if two papers from these subdisciplines are cited together in a third paper. Co-citations measure the contextual similarity between two references [19] as “the degree of relationship or association between papers as perceived by the population of citing authors” (p. 265). If aggregated on the level of scientific disciplines and subdisciplines, it thus reflects the combination of resources from different (sub)disciplines by the authors of the paper. Co-cited subdiscipline pairs were extracted from the reference lists of citing papers using a binary approach, i.e., counting each pair once per citing paper, irrespective of the number of papers associated with the particular subdiscipline in the reference lists of the citing paper. Hence, if a paper A cited five references in ophthalmology, three in astrophysics, and ten in zoology, then three co-citation links were created based on paper A: ophthalmology-astrophysics, ophthalmology-zoology and astrophysics-zoology. The frequency of occurring subdiscipline pairs equals the number of papers co-citing papers from the two particular subdisciplines. Each co-cited interdisciplinarity pair was assigned its distance on the UCSD map to represent its topical proximity. Pairs were grouped into 10 categories of distances (A-J) with a comparable number of pairs in each category.

The dataset used in this paper is drawn from Thomson Reuters’ WoS database, including the Science Citation Index Expanded (SCIE), the Social Sciences Citation Index (SSCI) and the Arts and Humanities Citation Index (AHCI). It comprises 11.1 million papers (articles and reviews) published between January 1, 2000 and December 31, 2012 and their cited references matched to the UCSD classification system via journal name. Only references from this period were considered, as the UCSD classification system builds on 2000-onwards publications. This leads to a lower proportion of references covered for the older years, and to a higher proportion for the more recent ones. Given that the analysis is based on the entire 13-year period instead on an annual basis, the different coverage of the specific years should not affect the results. References were linked to their record as a source item using Thomson’s ItemID_ref as well as in-house matching using the first author, publication year, volume number, and page number. The match of cited references with the source item allows assigning a discipline and subdiscipline to each cited reference. We excluded the 40 journals that are fractionally assigned to more than one UCSD subdiscipline to avoid artificially creating subdisciplinary pairs due to double classification. Based on this assignment, a paper is defined as interdisciplinary if it contains references from more than one subdiscipline. 17.2% of the 11 million papers in the database cited references of one subdiscipline only.

On the whole, 70.0% of all references made were matched to source items indexed in the WoS, 45.8% of which were published in the period analyzed. A total of 138,657,550 or 32.1% of all cited references were matched to a UCSD subdiscipline and were taken into account for the calculation of citation impact of co-cited interdisciplinary pairs. The percentage of references matched to source items and published between 2000 and 2012 varied among disciplines, given the different use of journal literature [20,21] and age of references. The percentage of references made to WoS-indexed items was highest in *Biotechnology* (84.6%) and lowest in *Humanities* (7.0%) on the level of disciplines and if limited to the publication years under analysis, highest in *Infectious Diseases* (39.9%) and *Brain Research* (39.6%) and lowest in *Social Sciences* (16.9%) and in the *Humanities* (2.4%). At the higher end of the spectrum for subdisciplines, more than 50% of references were matched with a source item and published between 2000 and 2012 in *Molecular Biology Methods* (56.2%), *Nanotechnology* (54.8%), *Proteomics* (54.1%), *Cancer* (*translated*; 54.0%) and *Clinical Medicine* (*translated*; 52.15), while this percentage remained below 1% in *Literary Criticism*, *Hispanic Studies*, *Italian Studies*, *Art History*, *Textile Art*, *Medieval History*, and *Modern Language*.

The final dataset of interdisciplinary papers contained a total of 9,166,710 papers and their cited references matched to a UCSD subdiscipline. All unique subdiscipline pairs (see binary definition above) were extracted from each of the papers. A threshold of 30 co-citing papers at the level of subdisciplines during the 2000–2012 publication period was applied to assure a minimum robustness of citation rates. Of the 153,181 possible pairs of the 554 UCSD subdisciplines, 80,997 (52.9%) were co-cited by at least 30 papers 179 million times amounting to a mean number of 2,214 co-citing papers per subdiscipline pair. As to be expected, the number of co-cited papers per interdisciplinary pair is positively skewed and the average number of co-citations per pair decreases continuously per distance categories A to J, i.e., the more distant the two disciplines the smaller the number of co-citing papers.

**Fig 6:**
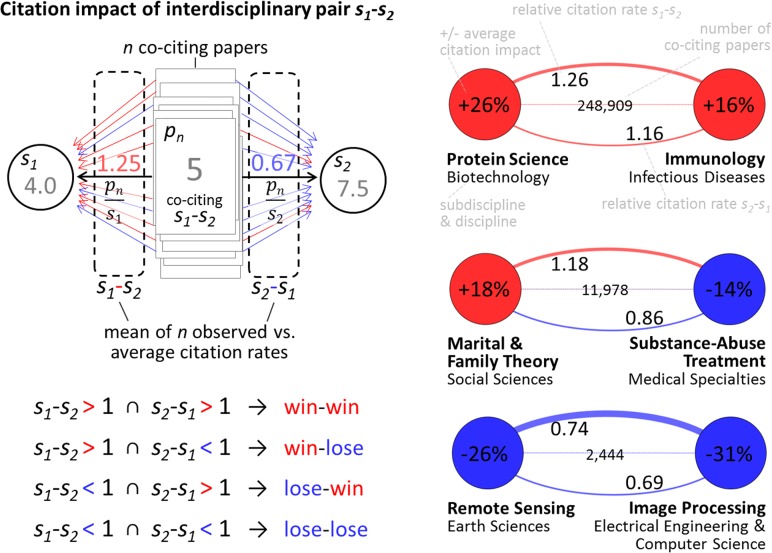
Schematic representation of the calculation and definition of citation impact of interdisciplinarity pairs.

Two normalization methods are performed in this paper. A first one, which is used in Fig 1, takes the subdiscipline (and year) of the journal in which the paper is published as the denominator, and then divides each paper’s observed number of citations by this expected value. In this case, each paper only has one normalized citation rate, and a clear zero-sum game at level of all paper is observed. A second one, used to measure the impact of interdisciplinary relationships, is performed at the level of the paper and subdiscipline combination. In this case, each pair obtained two relative, i.e., observed vs. expected, citation scores normalized by the average citation rates of all papers—interdisciplinary or not—that cited each of the two subdisciplines. Citations of each citing paper were counted from for the entire period studied, 2000–2013. This normalization procedure was applied to account for the fact that the citation impact of interdisciplinary papers depends on the citation potential of the involved subdisciplines [10]. The impact of a paper p co-citing subdisciplines *s*_*1*_ and *s*_*2*_ needs to be calculated in the citation context of both *s*_*1*_ and *s*_*2*_ separately. The observed citation score of *s*_*1*_ *–s*_*2*_ represents the citation score of *p*. The expected citation rates for each co-cited subdiscipline are based on the citation rate of all papers citing the particular subdiscipline in the same publication year to normalize for age. In this normalization approach, the expected citations of the subdiscipline are defined by its direct citation environment. For subdisciplines *s*_*1*_ and *s*_*2*_ co-cited by paper *p* in year *y*, the expected citation rate of subdiscipline *s*_*1*_ is thus the average citation rate of all papers citing subdiscipline *s*_*1*_ in year *y*. The relative citation rate of the interdisciplinary pair *s*_*1*_ *–s*_*2*_ relative to *s*_*1*_ equals the average of all observed vs. average ratios on the document level of all co-citing papers in all years, conforming to the average-of-ratio method [22]. The relative citation rate of the interdisciplinary pair *s*_*1*_ *–s*_*2*_ relative to *s*_*1*_ is based on the expected citations for *s*_*1*_. Two distinct relative citation rates were computed for the 80,997 occurring subdiscipline pairs, resulting in an asymmetric matrix with 161,994 relative citation rates. Thus, each co-cited pair *s*_*1*_ *–s*_*2*_ obtained two distinct observed vs. expected citation rates, normalized by the citation rates of papers that cited the subdiscipline *s*_*1*_ and *s*_*2*_, respectively, in the same year. Based on the pairwise comparison of the relative citation rates of co-cited subdisciplines, the success of interdisciplinary relationships was defined as win-win, win-lose/lose-win and lose-lose citation outcomes. If, on average, papers co-citing subdiscipline pair *s*_*1*_ *–s*_*2*_ were cited above expectations in both *s*_*1*_ and *s*_*2*_ the interdisciplinary relationship *s*_*1*_ *–s*_*2*_ was defined as a win-win relationship in terms of observed vs. expected citation impact, while a lose-lose situation was at hand if it did not exceed expected citation impact in both *s*_*1*_ and *s*_*2*_. If citation impact was above expectations compared to *s*_*1*_ but below average compared to *s*_*2*_ or below *s*_*1*_ and above *s*_*2*_, the interdisciplinary relationship *s*_*1*_ *–s*_*2*_ was defined as a win-lose or lose-win connection, respectively, see Fig 6 and examples therein. While a zero-sum can be obtained here at the level of paper and cited subdisciplines combinations, it cannot be observed for subdiscipline pairs, as papers citing *n* subdisciplines appear *n* ^*2*^ *-n* times (because the diagonal is empty) and each subdiscipline pair is based on a different number of papers. While the first normalization method is used to show the scientific impact of papers citing a certain number of subdisciplines (i.e. level of interdisciplinarity), the second is used to assess the scientific impact co-cited subdisciplines.

Success of interdisciplinarity in terms of citation impact is visualized using a heat map (Fig 4) and network graphs (Fig 5). The heat map shows the mean relative citation impact of subdiscipline pairs, i.e., each cell of the matrix represents the mean relative citation impact of a particular pair. The asymmetrical square matrix contains 554 rows and columns representing the subdisciplines of the classification, which are grouped according to the 13 superordinate disciplines. Cells representing subdiscipline pairs with citation rates above world average are indicated by shades of red, those below by shades of blue, while citation rates close to world average are colored in white. About half of the matrix cells remain empty because the respective interdisciplinary pair did not meet the threshold of 30 co-citing papers or they were strictly disciplinary (i.e., the diagonal of the matrix). The empty cells are also colored in white and can thus not be distinguished from cells with citation impact close to world average. Hence, the heat map highlights the winners and losers of interdisciplinary relationships from the perspective of both *s*_*1*_ and *s*_*2*_.

While the heat map (Fig 4) represents the 161,994 observed vs. expected citation impacts of interdisciplinary relationships of subdiscipline pairs, the win-win and lose-lose relationships with the highest citation impact are visualized in the UCSD map of science (Fig 5) in order to show which interdisciplinary combinations are most successful and to communicate the distances of the subdisciplines involved, see Fig 5 A4 and B4.
